# Natural autoantibodies to the gonadotropin-releasing hormone receptor in polycystic ovarian syndrome

**DOI:** 10.1371/journal.pone.0249639

**Published:** 2021-04-02

**Authors:** Lisa-Marie Sattler, Hanna A. Schniewind, Waldemar B. Minich, Christoph W. Haudum, Petra Niklowitz, Julia Münzker, Gábor L. Kovács, Thomas Reinehr, Barbara Obermayer-Pietsch, Lutz Schomburg

**Affiliations:** 1 Institute for Experimental Endocrinology, Charité-Universitätsmedizin Berlin, Berlin, Germany; 2 Corporate Member of Freie Universität Berlin, Humboldt-Universität zu Berlin, and Berlin Institute of Health, Berlin, Germany; 3 Department of Internal Medicine and Department of Gynecology and Obstetrics, Endocrinology Lab Platform, Medical University Graz, Graz, Austria; 4 Center for Biomarker Research in Medicine (CBmed), Graz, Austria; 5 Department of Pediatric Endocrinology, Vestische Kinder- und Jugendklinik, University of Witten-Herdecke, Datteln, Germany; 6 Integrated Research and Treatment Centre for Adiposity Diseases, Department of Medicine, University of Leipzig, Leipzig, Germany; 7 1st Department of Internal Medicine, Flor Ferenc Hospital, Kistarcsa, Hungary; Lewis Katz School of Medicine at Temple University, UNITED STATES

## Abstract

**Context:**

Polycystic ovarian syndrome (PCOS) is a complex disease with different subtypes and unclear etiology. Among the frequent comorbidities are autoimmune diseases, suggesting that autoantibodies (aAb) may be involved in PCOS pathogenesis.

**Objective:**

As the gonadal axis often is dysregulated, we tested the hypothesis that aAb to the gonadotropin-releasing hormone receptor (GnRH-R) are of diagnostic value in PCOS.

**Design:**

An in vitro assay for quantifying aAb to the GnRH-R (GnRH-R-aAb) was established by using a recombinant fusion protein of full-length human GnRH-R and firefly luciferase. A commercial rabbit antiserum to human GnRH-R was used for standardization. Serum samples of control subjects and different cohorts of European PCOS patients (n = 1051) were analyzed.

**Results:**

The novel GnRH-R-aAb assay was sensitive, and signals were linear on dilution when tested with the commercial GnRH-R antiserum. Natural GnRH-R-aAb were detected in one control (0.25%) and two PCOS samples (0.31%), and 12 samples were slightly above the threshold of positivity. The identification of samples with positive GnRH-R-aAb was reproducible and the signals showed no matrix interferences.

**Conclusion:**

Natural GnRH-R-aAb are present in a very small fraction of adult control and PCOS subjects of European decent. Our results do not support the hypothesis that the GnRH-R constitutes a relevant autoantigen in PCOS.

## Introduction

Fertility constitutes an essential issue for both adult males and females. It is estimated that 3.5% to 16.7% of women in developed nations are suffering from infertility, with more than half of them seeking medical care [1]. One of the frequent underlying reasons for female infertility is polycystic ovarian syndrome (PCOS), affecting about 5% to 15% of women of reproductive age [2–5]. PCOS is a chronic and complex disease with a rising incidence in most populations, making PCOS one of the most common endocrine-metabolic disorder in adult women [6]. The clinical presentation is heterogeneous and established pathognomonic serological markers are missing, hindering a fast and reliable diagnosis [7,8]. Menstrual disturbances such as cycle irregularities are among the first symptoms, and signs of virilisation like hirsutism, a deep voice, acne or alopecia may develop [9,10]. The current diagnosis is based on the Rotterdam criteria from 2003 [3,11], i.e., when any two of the following three criteria are met; i) clinical or biochemical diagnosis of hyperandrogenism, ii) oligo- or amenorrhea (or rather oligo- or anovulation), and/or iii) morphological presence of polycystic ovaries by ultrasound inspection (presence of ≥12 follicles of 2–9 mm in diameter and/or ovarian volume ≥10 ml).

However, around one half of the affected women are on a year-long journey with three or more specific health professionals needed for establishing the diagnosis, causing dissatisfaction and disappointment with the current medical care and information on PCOS [7]. During diagnosis, other reasons potentially leading to irregular menstruation or high androgen levels have to be excluded, e.g. M. Cushing, non-classical congenital adrenal hyperplasia, androgen-producing tumors or hyperprolactinemia [12]. Still, the diagnosis is challenging and the criteria are on debate as more than 50% of patients display signs of insulin resistance, which may occur independently of obesity [4,5], and may promote additional comorbidities like diabetes mellitus, dyslipidemia or hypertension [12,13]. Collectively, hyperandrogenism and insulin resistance seem to constitute the chief culprit of PCOS, calling for an update and respective modification of the diagnostic criteria used in the clinical routine workup of symptomatic women [14].

In general, about 75% of PCOS women are overweight and about 70% show dyslipidemia, contributing to a relatively high mortality rate due to cardiovascular events [15]. Furthermore, affected women have an increased risk for autoimmune diseases, including Hashimoto’s thyroiditis, Graves’ disease and diabetes mellitus type I [4,15,16]. There are reports on the presence of certain autoantibodies (aAb) known from other autoimmune diseases in PCOS, e.g. aAb to dsDNA, histones, TPO, islet-cells or SMTRNP [16,17].

No causal therapy is currently available. In many cases, weight loss serves as a first promising and meaningful measure, as it improves insulin sensitivity, hyperandrogenemia and irregular menstrual cycles [13,18]. The administration of oral contraceptives similarly contributes to restoring the monthly rhythm and mitigating hyperandrogenemia [4], but is limited as therapeutic tool for women without the intention to become pregnant. Metformin may improve glucose handling, which also lowers androgen levels and positively affects fertility [4]. In case a patient nevertheless stays anovulatory, gonadotropins in combination with GnRH may be applied [19]. Other supportive medication include antiandrogens, glucocorticoids, isotretinoin or statins in addition to metformin, bariatric surgery or certain micronutrients such as vitamin D, which has been reported to improve ovulatory function and hyperlipidemia [4,15]. Another promising approach for positively affecting weight and fat mass would also include alteration of the gut microbiome by dietary means using prebiotics, probiotics or synbiotics [20].

Collectively, these diverse treatment options highlight the variable clinical picture of PCOS, the missing understanding of disease etiology and the urgent need for better prognostic and diagnostic biomarkers. A recent report suggested the existence of aAb to the second extracellular loop of the gonadotropin-releasing hormone receptor (GnRH-R) in the majority of PCOS serum samples tested [21]. The GnRH-R belongs to the family of G protein-coupled receptors (GPCR) and is inserted into the plasma membrane of GnRH target cells, with high expression in gonadotropic cells of the anterior pituitary. It controls biosynthesis of the anterior pituitary glycoprotein-hormones luteinizing hormone (LH) and follicle-stimulating hormone (FSH), exists in different variants, and associates with G-proteins that activate the IP3-calcium and cAMP-PKA second messenger signaling cascades [22]. GnRH and the GnRH-R are central components of the endocrine axis controlling the ovary, and dysregulation of either the hypothalamic ligand or the pituitary receptor are associated with impaired fertility [23]. Autoimmunity to the GnRH-R would constitute a plausible underlying mechanism of dysregulation, given that GnRH controls the gonadotropins FSH and LH that are dysregulated in PCOS [24]. It is well established that autoantibodies to G-protein coupled receptors (GPCR) are capable of disturbing endocrine signaling, e.g. in Graves’ [25] or Chagas disease [26]. An autoimmune origin of the disturbed gonadal axis is also supported by the notion that autoimmune diseases like Hashimoto’ thyroiditis or systemic lupus erythematosus are more prevalent in females with PCOS than in the general population [16]. To verify this hypothesis experimentally, we have established a novel and highly sensitive assay for detecting and quantifying aAb to human GnRH-R (GnRH-R-aAb) by using the full-length human G protein-coupled receptor (GPCR). The assay’s properties were thoroughly characterized and GnRH-R-aAb prevalence was compared in healthy subjects and different cohorts of women with PCOS from three European countries. Our results indicate that GnRH-R-aAb are a rare finding and likely not of relevance for general PCOS risk assessment or diagnosis.

## Materials and methods

### Human samples

A set of high quality serum samples from adult subjects (n = 400, 50% female, self-reported status as healthy) was purchased from a commercial supplier (in.vent Diagnostica GmbH, Hennigsdorf, Germany), assuming that a group size of n = 200 female and n = 200 male samples will be of sufficient size to determine the prevalence of GnRh-R-aAb in the normal population background and define the technical background noise of the novel aAb assay. Three different cohorts of PCOS were analyzed, comprising a set of 651 serum samples. Samples were collected from female patients in Graz, Austria (PCOS-A, n = 576, 78.5% PCOS), from adolescent patients in Datteln/Witten/Herdecke, Germany (PCOS-DE, n = 40, 50.0% PCOS), and from patients in Budapest, Hungary (PCOS-HU, n = 35, 68.6% PCOS). Written informed consent for participation was obtained from all patients included, or from one or both of their parents in case the participants were below 18 years of age (all subjects in PCOS-DE). No particular selection with regard to any genetic or environmental factors was made. An overview on anthropometric characteristics is provided, highlighting that PCOS-A was contributing the largest cohort without preselection of anthropometric or clinical characteristics, PCOS-DE was exclusively enrolling adolescent PCOS patients, whereas PCOS-HU constituted a small set of predominantly young and obese patients (**Table 1**). Diagnosis of PCOS was based on clinical presentation and disease manifestation according to the Rotterdam criteria [3,11]. The androgen status of patients enrolled in the PCOS-A cohort was assessed routinely by the analysis of several steroid hormones, including total testosterone (total Testost.), free testosterone (free Testost.), dehydroepiandrosterone-sulfate (DHEAS) and androstenedione. Total Testost. was quantified by ELISA (ADVIA Centaur® Immunoassay, Siemens Healthcare Diagnostics Inc., Tarrytown, NY, USA), whereas free Testost., DHEAS and androstenedione were measured using liquid chromatography/mass spectrometry (LC/MS) as described [27].

**Table 1:** Characterization of the cohorts of patients studied in this analysis.

Cohort	PCOS-A*	PCOS-DE	PCOS-HU
City, Country	Graz, Austria	Datteln, Germany	Budapest, Hungary
Samples, total [n]	**576**	**40**	**35**
PCOS diagnosis [n]	**452**	**20**	**24**
Age (median, IQR) [y]	**26.8 (23, 30)**	**15.1 (14.6, 16.0)**	**23.0 (21.0, 29.8)**
BMI (median, IQR) [kg/m^2^]	**26.3 (21.4, 30.5)**	**36.0 (31.0, 41.2)**	**27.8 (22.8, 38.6)**

* >99% with Caucasian ethnicity, i.e., European subjects from Styria, Austria.

The investigation was conducted in accordance with the guidelines in the Declaration of Helsinki, informed consent of the patients or parents was obtained, and the samples were analyzed in a blinded manner at a lab distant from the clinical sites. The analyses have been approved by the ethical review committees of the universities in Graz (Ethikkommission der Medizinischen Universität Graz, LKH-Universitätsklinikum, Austria; EC18-066 ex 06–07), Datteln/Witten/Herdecke (Ethik-Kommission der Universität Witten/Herdecke e.V., Witten, Germany; EV No. 15/2006) and Budapest (University Ethics Committee, Semmelweis University, Budapest, Hungary; ETT-TUKEB 42506-1/2016/EKU).

### Construction of GnRH-R luciferase fusion protein

The open reading frame of human GnRH-R was synthesized by Eurofins Genomics (Ebersberg, Germany) and used as template for cDNA cloning. DNA primers used for amplification were synthesized by BioTeZ (Berlin, Germany). pSP-Luc+NF vector was obtained from Promega (Promega, Mannheim, Germany, catalog Nr. #E4471), pIRESneo vector from Addgene (Addgene, Teddington, UK, catalog Nr. #6988–1), and a polyclonal rabbit IgG antiserum to human GnRH-R was purchased from Thermo Fisher (rabGnRH-R-Ab, Thermo Fisher Scientific, Schwerte, Germany, catalog Nr. #PA5-33597). In order to construct a dilution series of positive samples, the commercial polyclonal antiserum to human GnRH-R was diluted stepwise in human control serum.

The expression vector encoding human GnRH-R fused in frame to firefly luciferase (Luc) was constructed by first amplifying the cDNA of firefly luciferase using pSP-Luc+NF as template and inserting it into plasmid pIRESneo generating pIRESneo-Luc. The amplified cDNA of human GnRH-R was inserted N-terminal to Luc giving rise to plasmid pIRESneo-GnRH-R-Luc encoding the GnRH-R-Luc fusion protein. The correct reading frame of the recombinant fusion protein was verified by DNA sequencing of pIRESneo-GnRH-R-Luc using a service provider (LGC Genomics GmbH, Berlin, Germany).

### Stable expression of GnRH-R-Luc in mammalian cells

Human embryonic kidney cells (HEK 293 cells) were grown in DMEM containing 10% fetal bovine serum (FBS) and transfected with pIRESneo-GnRH-R-Luc using FuGENE6 reagent (Promega, catalog Nr. #E2691). Stable cell clones were selected by adding G418 (0.8 mg/ml, Sigma-Aldrich, catalog Nr. #A1720), and the resulting Luc activity of established cultures was compared. Aliquots of cells were lysed and tested for Luc activity and compared, as the expression levels of the transgene differ between the clones depending on the particular site of integration into the host genome. The most productive clone was expanded in DMEM/FBS/G418 until confluency in 100 mm-cell-culture-dishes, and cells were collected by scraping. The suspension was centrifuged (Megafuge 1.0R, Heraeus, Hanau, Germany) at 3500 rpm (2000 g) at 4°C for 10 min, and washed twice with PBS. After removing the supernatant, the cell pellet was resuspended in a buffer containing 50 mM Tris HCl (pH 7.4), 100 mM NaCl and 10% glycerol. Finally, Triton X-100 was added (to 10% f.c.) to lyse the cell membranes. After an incubation step of 5 min on ice and another centrifugation step (3500 rpm, 2000 g, 10 min, 4°C), the supernatant was collected and stored at -80°C until it was used in the GnRH-R-aAb assay. A control preparation with recombinant firefly luciferase alone, not containing the human GnRH-R attached, was prepared and tested under the same conditions as above, in order to verify absence of antibodies recognizing the reporter enzyme.

### Immunoprecipitation for quantification of GnRH-R-aAb

The GnRH-R-Luc cell extract was diluted 10-times with buffer containing 50 mM Tris (pH 7.5), 100 mM NaCl, 10% glycerol, 5% milk powder, 5% glucose, 1% Triton X-100 and 0.01% sodium azide. To prepare measurements, 96-well-plates were cooled on ice and pre-loaded with 40 μl of the GnRH-R-Luc extract. Then, 10 μl of pre-diluted serum (1:1, vol/vol, with dilution buffer consisting of 50% glycerol, 100 mM NaCl and 50 mM Tris HCl, pH 7.4) were added and incubated overnight at 4°C in order to allow formation of immune complexes between GnRH-R-specific aAb and the GnRH-R-Luc fusion protein. The following day, 40 μl protein A-Sepharose suspension (Merck KGaA, Darmstadt, Germany, catalog Nr. #GE17096303) was added and incubated for one hour at room temperature. The 96-well-plates were washed and centrifuged six times with 200 μl washing buffer containing 50 mM Tris HCl (pH 7.4), 100 mM NaCl, 10% glycerol and 0.5% Triton X-100. The supernatant was finally aspired by vacuum and the pellets were resuspended in luciferase substrate buffer containing 0.5% Triton X-100, and then transferred into glass tubes. Luciferase activity was measured in a chain luminometer (Berthold, Bad Wildbad, Germany) for 5 sec with 200 μl luciferase substrate buffer per tube. Relative light units (RLU) were recorded and analyzed as described [28].

### Statistical analysis

Statistical analyses were performed using GraphPad Prism v.7.0 (GraphPad Software Inc., San Diego, USA) or SPSS (version 25, SAS Institute, Cary, NC, USA). Normal distribution was assessed by the Shapiro-Wilk–Test, and quantitative variables were compared by unpaired t test (normal distribution). Alternatively, data were compared by two-sided non-parametric U Mann-Whitney Test. Results are shown as mean ± SD or median with interquartile range (IQR). Statistical significance is assigned as *, P<0.05, **, P<0.01 or ***, P<0.001.

## Results

### GnRH-R expression and generation of GnRH-R-aAb assay

**Fig 1:**
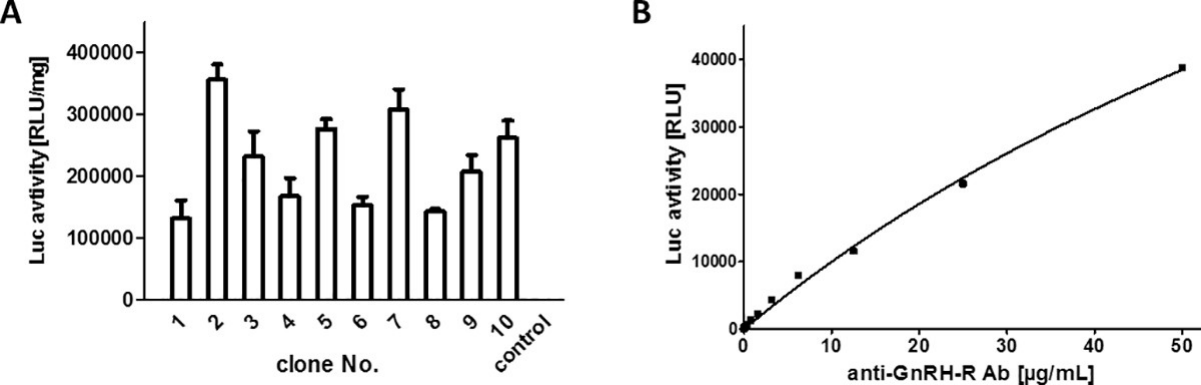
Generation of stable clones and linearity of the assay for GnRH-R-aAb quantification. A) Stable cultures of HEK293 cells expressing the recombinant fusion protein were established and ten productive clones were compared for luciferase (Luc) activity, which is indicative of the relative concentration of the desired GnRH-R-Luc fusion protein. B) A commercial polyclonal rabbit antiserum to human GnRH-R (anti-GnRH-R Ab) was diluted and tested in the newly generated assay. The signals obtained were related to the anti-GnRH-R Ab, and a proportional response of signal intensity to the antiserum concentration was recorded. Measurements were conducted in duplicates, control samples without human serum typically yield luminescence signals of <100 RLU.

Human HEK293 cells were transfected with pIRESneo-GnRH-R-Luc and stable clones were selected by applying G418 as selection antibiotic. Aliquots of cells were lysed and tested for Luc activity. Several positive clones were isolated, propagated and used to generate stable cell stocks for further use (**Fig 1A**). The clone with highest expression was expanded and the GnRH-R-Luc fusion protein was isolated and tested with the commercial antiserum raised to human GnRH-R in rabbit (rabGnRH-R-Ab). Protein A-mediated precipitation of rabGnRH-R-Ab incubated with extracts from HEK293 cells expressing pIRESneo-GnRH-R-Luc but not with extracts from untransfected HEK293 control cells yielded high Luc signals (expressed as relative light units, RLU). The signals of the precipitated protein A-bound complexes between GnRH-R-Luc and the commercial rabGnRH-R-Ab were linear on dilution over a wide concentration range (**Fig 1B**).

### Prevalence of GnRH-R-aAb in PCOS versus controls

**Fig 2:**
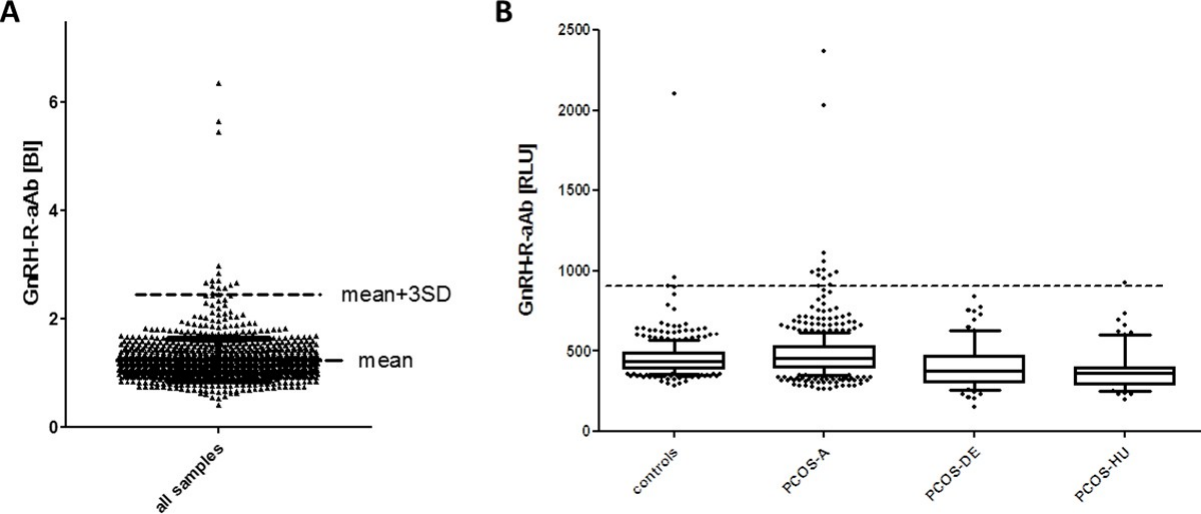
Prevalence of GnRH-R-aAb in serum samples of controls and PCOS patients. A) The full set of samples (n = 1051) was analyzed for GnRH-R-aAb concentrations by measuring GnRH-R-Luc activity in the immunoprecipitates. Binding indices (BI) were calculated based on the lower half of all samples. The mean value plus three standard deviations (mean+3SD) was used as criterion for outliers, indicating positive presence of GnRH-R-aAb. Three samples were strongly positive, and 12 samples were slightly above the threshold (dashed line). B) The signals for GnRH-R-aAb were not normally distributed in the cohorts of samples analyzed. The prevalence of highly positive samples is similar in the cohorts of PCOS patients and control subjects (2 out of 651, i.e., 0.31%, vs. 1 out of 400, i.e., 0.25%).

In order to test for natural GnRH-R-aAb in PCOS, a total of >1000 human serum samples were analyzed by the newly generated assay (**Fig 2**). Most of the signals obtained were in a close range of 250 to 750 RLU. According to the Shapiro-Wilk normality test, the values are not normally distributed (W = 0.7133, P<0.0001). The threshold for positivity of GnRH-R-aAb was calculated on basis of the bottom half of all signals, and set at the sum of the mean value plus three standard deviations (mean+3SD). The binding index (BI) denotes the factor above the mean. By applying this criterion, we identified a total set of 15 samples as being positive for containing GnRH-R-aAb (**Fig 2A**). However, the majority of which (n = 12) is close to the threshold (BI = 2.4), leaving a set of three samples only as strongly positive, displaying BI of 5.46, 5.65 and 6.36, respectively. Specificity of the three highly positive serum samples for binding to the GnRH-R moiety of the luciferase-fusion protein detector was verified by analysis with an unmodified luciferase as reporter, yielding background signals only.

Comparing the different cohorts of samples tested, the positive samples were identified in the set of healthy controls (n = 2, with one slightly and one strongly positive signal), in the large cohort of adult PCOS patients from Graz (PCOS-A, n = 12, with two strongly positive samples), and one sample in the small cohort of Hungarian PCOS patients (PCOS-HU). There was no positive sample in the group of young adolescent PCOS patients (PCOS-DE) (**Fig 2B**). A direct comparison of samples strongly positive for GnRH-R-aAb in relation to PCOS indicates a similar prevalence in controls and patients, with 0.25% (1/400) in the cohort of self-reported healthy controls, and 0.31% (2/651) in the cohorts of PCOS patients, respectively.

### Stability of GnRH-R-aAb in positive serum samples

**Fig 3:**
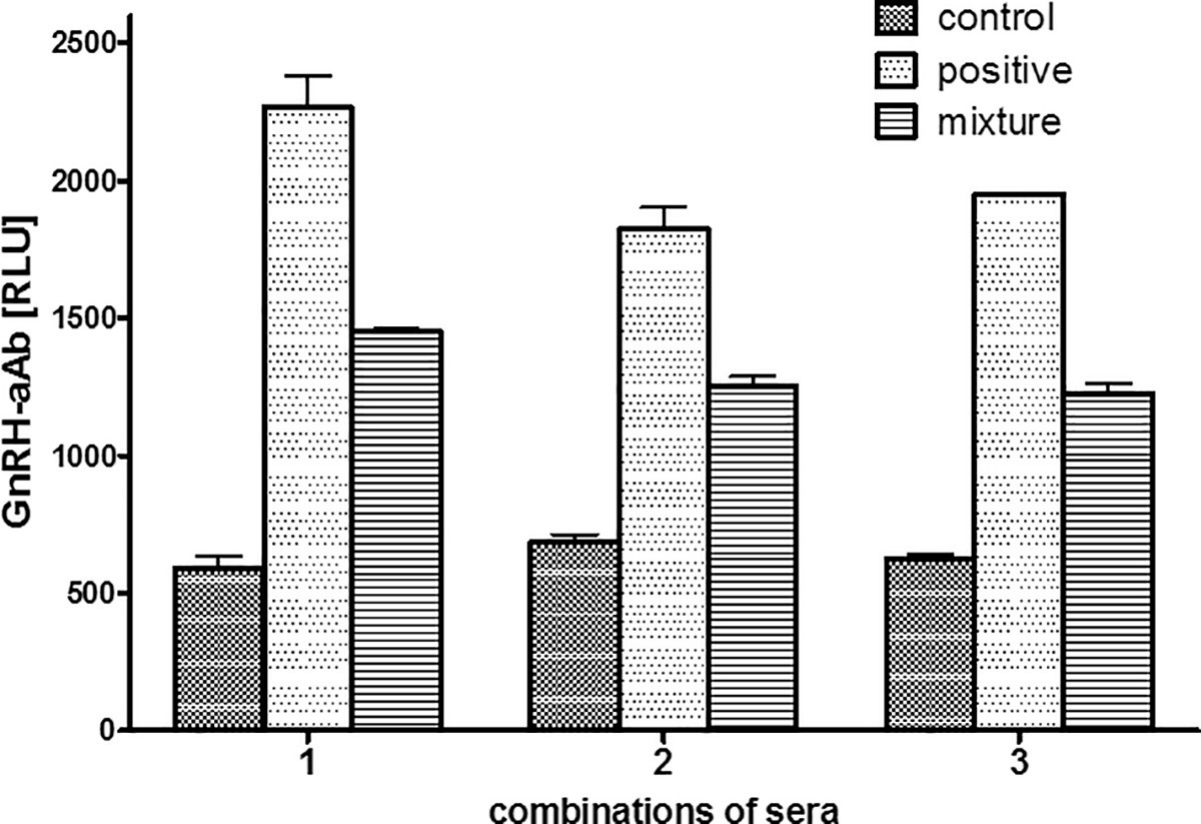
Test for matrix effects and signal reproducibility. Three different control samples were equally mixed with one of the three highly positive GnRH-R-aAb samples and analyzed for GnRH-R-Ab concentrations. The signals measured correspond to the theoretical arithmetic mean of the respective serum samples indicating the absence of matrix effects. Measurements were conducted as duplicates.

The results were reproducible, and the same three sera (one control and two PCOS; #175 and #225) were verified as strongly positive in a second round of measurements. All three sera were recognizing the GnRH-R moiety of the detector, and showed no binding to luciferase without the fusion partner. In order to test for stability of signals and potential interference by matrix, analyses of mixed serum samples were conducted. To this end, a positive and a negative sample each were combined (1:1, vol/vol) and measured as described above, i.e., one set of test tubes contained 5 μl of a negative serum sample (control), another contained 5 μl of one of the three positive serum samples (positive), and the third contained 5 μl of a mixed serum, consisting of a 1:1 mixture (vol/vol) of the control and the positive serum samples (mixture). The results indicate that the resulting signal from the mixture was equal to the arithmetic mean of the signals from the negative control and the positive GnRH-R-aAb containing serum, highlighting assay reproducibility, and excluding major matrix effects on the signals recorded in the GnRH-R-Ab assay **(Fig 3**).

### Steroid hormone levels in PCOS patients with GnRH-R-aAb

**Fig 4:**
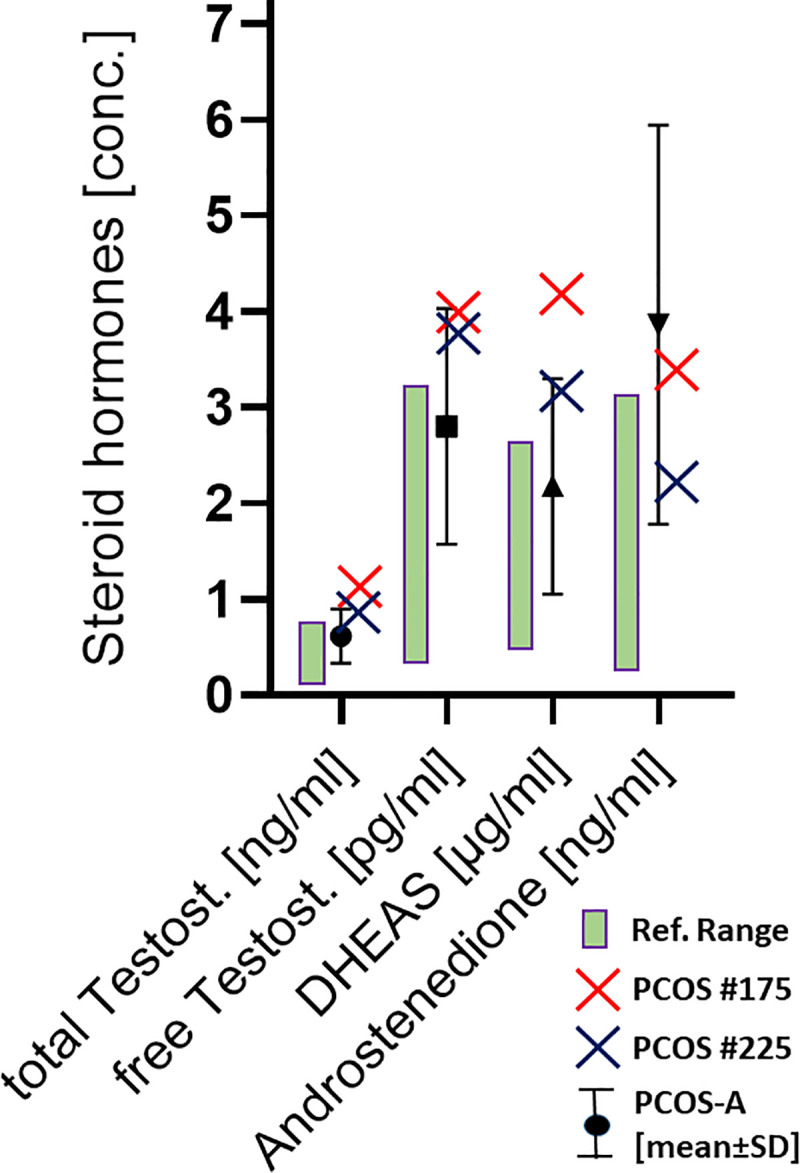
Steroid hormone levels in the two patients with high GnRH-R-aAb concentrations in comparison to healthy controls and other PCOS patients. Two adult female PCOS patients were identified as highly positive for GnRH-R-aAb (PCOS #175; red cross, #225; blue cross). Both patients displayed elevated androgen concentrations in comparison to the reference range of healthy adult women (Ref. Range, green bar), and also in comparison to the other samples from the same cohort of PCOS patients (PCOS-A) (mean +/-SD; black symbols with error bars).

The GnRH-R on pituitary gonadotrophs controls LH and FSH biosynthesis and secretion and thereby affects steroid hormone status. In order to test for a potential effect of GnRH-R-aAb on circulating steroid hormones, serum concentrations of total Testost., free Testost., DHEAS and androstenedione were determined in GnRH-R-aAb positive PCOS patients and compared to the reference range of healthy women and to the range of the other adult patients of PCOS-A. The direct comparison indicates that both patients with positive GnRH-R-aAb (PCOS #175 and #225) were characterized by hyperandrogenemia in comparison to healthy women (Ref. Range). In particular, the patient with the highest GnRH-R-aAb concentrations (PCOS #175) displayed not only elevated androgen levels in comparison to the Ref. Range, but also relatively high concentrations of total testosterone and DHEAS as compared to the other adult PCOS patients (**Fig 4**). The second positive PCOS patient with GnRH-R-aAb (PCOS #225) showed the same pattern of elevated steroids as PCOS #175, albeit less pronounced.

## Discussion

In this explorative study, we tested the hypothesis of a high prevalence and considerable diagnostic value of GnRH-R-aAb in PCOS. The notion was based both on endocrine [24] and genetic [29] considerations as well as on a recent report describing high prevalence of GnRH-R-aAb in PCOS sera using an extracellular loop peptide as antigen [30]. In order to generate a reliable and scalable diagnostic assay of high sensitivity, we decided to use as antigen a receptor fusion protein with firefly luciferase (GnRH-R-Luc). In view that the extracellular N-terminus of human GnRH-R is only 34 amino acids in length, and none of the three extracellular loops are any longer, we decided to using the full-length receptor expressed in a human cell line as bait. Choosing the intracellular C-terminus as fusion site, we expect all extracellular domains of the fusion protein to become available to potential GnRH-R-aAb, including the N-terminus and all the extracellular loops. In analogy to our experiences with the TSH-receptor, we expected hereby to enable the detection of both linear and three-dimensional epitopes, respectively, the latter of which are likely to be missed when using small linear peptides of a certain region only [31]. Our results indicate that indeed a few European subjects express natural GnRH-R-aAb, and that the receptor-specific aAb can reproducibly be detected and quantified.

In our study, we were also successful in identifying a suitable positive standard antiserum that recognizes human GnRH-R reliably, and that can be used by other research teams for independent studies on this issue. Importantly, the rabbit antibodies used as standard are also efficiently recognized by protein A during immunoprecipitation, as expected from its ability to bind IgG from different species [32]. However, in our study, the prevalence of natural GnRH-R-aAb was very low and not different between healthy controls and European PCOS patients. This finding indicates that the GnRH-R can be recognized as an antigen, but that it does not constitute a relevant diagnostic parameter in PCOS, in contrast to a recent report [30]. Even our newly developed assay with full-length human GnRH-R expressed in human cells as bait for GnRH-R-aAb seems not to be of diagnostic value for predicting PCOS risk or accelerating diagnosis. Nevertheless autoimmunity to GnRH-R does happen and might develop in selected cases to an extent that may become of pathological relevance, even though such rare cases were not present in our analysis. Even though the two PCOS patients identified as positive for GnRH-R-aAb displayed a particular pattern of circulating androgens in comparison to control and to the other PCOS patients from the same cohort (PCOS-A), the prevalence of GnRH-R-aAb in PCOS was very low, the concentrations of the GnRH-R-aAb were not exceedingly high, and the deviation of the steroid levels from the range observed in PCOS is moderate. Collectively, we have to conclude that GnRH-R-aAb are very rare and not of general relevance for the diagnosis of PCOS in subjects with European ethnicity.

It is unfortunate, that the molecular pathogenesis of PCOS is not resolved and the phenotype is heterogeneous, hindering a fast and unequivocal diagnosis. PCOS presents as a highly multifactorial disease, where genetic predisposition, socioeconomic conditions, ethnicity, lifestyle and metabolic factors, as well as inflammatory and immunological reactions are causally involved and interact [12,33,34]. An autoimmune nature of PCOS still is a reasonable theory, in view that other autoimmune diseases and certain aAb are observed in PCOS with a higher frequency than in the general population [2,16,17]. It was thus unexpected and disappointing to identifying only few positive samples for GnRH-R-aAb in the full collection of samples tested, i.e., a total prevalence of 1.4% (15/1051) only, with just 0.3% being highly positive. Using the same technique of constructing an antigen receptor fusion protein with luciferase as reporter at the intracellular C-terminus, we identified around 10% of subjects positive for aAb to the IGF1-receptor (IGF1R-aAb) [28]. The IGF1R-aAb proved to inhibit growth hormone signaling and proliferation in vitro [28], and were associated with low muscle strength in adolescent subjects [35]. Due to the very small number of positive samples identified in the current study, a similar analysis on a potential physiological role of natural GnRH-R-aAb is not possible. A reason for the low prevalence of GnRH-R-aAb may be given in the nature of the protein as a GPCR, with only small portions of the protein exposed to the extracellular space, i.e., the N-terminus and the three small extracellular loops. This feature may have also contributed to the relatively low concentrations of the GnRH-R-aAb identified, barely surpassing the noise level in several of the statistically positive samples, leaving three samples only with GnRH-R-aAb concentrations in a considerable range that may be disease-relevant, as the disruptive effects of aAb on physiological or endocrine pathways usually correlates to aAb titer [36].

This low prevalence is in contrast to e.g. the TSH-receptor (TSH-R), which likewise belongs to the family of GPCR. Here, the extracellular domain is huge, and different aAb to the TSH-R are known capable of antagonizing TSH signaling or even of acting positively like TSH and causing constant stimulation leading to Graves’ disease and hyperthyroidism [37]. Until now, it appears that the complete full-length and correctly processed GPCR molecule is necessary as antigen to reliably measuring TSH-R-aAb, and smaller subdomains or peptides would not be suitable or sufficient [38]. More elaborate bioassays have recently been generated and described capable of discriminating the nature of the TSH-R-aAb as stimulating or not [39]. With the rare occurrence of GnRH-R-aAb detected in our study, the generation of a similar bioassay for the purpose of testing biological effects of these aAb appears not warranted at present.

The inherent complexity of reliably measuring aAb to members of the GPCR family as potential antigens in human disease is a well-recognized issue, and attempts to detecting e.g. aAb against the beta1-adrenergic receptor in Chagas’ disease by peptide-based assays failed until now [40,41]. A similar issue emerges when comparing the aAb prevalence reported for other membrane proteins, determined by peptide-based versus full-length protein assays, e.g. in case of the transporters for iodide. Here, peptide-based aAb assay have reported very high prevalence in different thyroid diseases, e.g. 84% of positive samples for the sodium-iodide symporter in Graves’ disease [42], or 97.5% positive samples for pendrin in Hashimoto’s disease [43]. These astonishing high values were not reproduced when full-length transporter molecules were used as antigens in radioligand binding assays [44], or when the transporters are used as antigens in the form of full-length fusion proteins with Luc [45]. Notably, the direct isotope labeling of the transporters and the Luc-based labeling yielded similar results, verifying the importance of full-length membrane proteins as bait in such analyses [44,45].

Our data do not argue against the notion of PCOS being an autoimmune disease in at least some patients, as there are many more relevant antigen candidates potentially affecting the gonadal axis and androgen concentrations, or the metabolic axes, body composition and insulin sensitivity. Prime candidates of the former are the pituitary receptors for LH and FSH, and comprehensive analyses for the potential involvement of aAb to these GPCR using full-length molecules as antigens are missing. Similarly, aAb to the insulin-receptor may be involved as they can affect insulin signaling along with metabolic control of the reproductive axis, and rare cases of high aAb levels to the insulin receptor have been described in ovarian overgrowth and hyperandrogenemia [46]. This hypothesis needs to be tested experimentally.

Among the strengths of our study are the high quality of the assay developed, its suitability for reproducible production and high throughput screening, and the demonstration of linearity of signals with a commercial antiserum as universally available standard. Moreover, the number of samples analyzed was sufficiently high to obtain a representative picture on the prevalence of natural GnRH-R-aAb in both healthy subjects and patients with PCOS. Finally, using full-length GnRH-R as antigen implies that all potentially relevant antigens were available and correctly processed to the sample tested, and it is unlikely that site-specific GnRH-R-aAb have escaped detection. Among the limitations of the study are the lack of knowledge on any potential clinical phenotype of the purchased set of healthy control samples, that we have analyzed cohorts of Caucasians only, and that we have not mapped the domain(s) recognized by the GnRH-R-aAb identified.

We conclude that the pathogenesis of PCOS remains poorly understood, and that the hypothesis of natural aAb to the GnRH-R constituting a frequent finding of predictive or diagnostic relevance for PCOS is not verified.

## Supporting information

S1 File (PDF)
