# Supplementary material for: Natural autoantibodies to the gonadotropin-releasing hormone receptor in polycystic ovarian syndrome
Source: PLoS One. 2021 Apr 2;16(4):e0249639. doi: 10.1371/journal.pone.0249639 (PMC8018624; doi:10.1371/journal.pone.0249639)
Supplement: S1 File — (PDF) [file pone.0249639.s001.pdf]

	1	2	3	4
RLU-1	102166	331089	191301	139275
RLU-2	161371	381587	273121	197159

5	6	7	8
292816	139279	341186	139275
261048	167151	275146	147150

9	10	control
234557	234550	71
180278	290277	79

Dilution	Serum 1
50	38790
25	21583
12,5	11593
6,25	8021
3,125	4401
1,563	2304
0,781	1301
0,391	754
0,195	391
0,098	286
0,049	208
0,024	151
0,012	190
0,006	121
0	111
0	106

controls	PCOS	all	all samples
283	441	283	0,4
295	383	295	0,52
299	467	299	0,55
307	377	307	0,57
311	660	311	0,61
325	623	325	0,63
326	363	326	0,63
328	381	328	0,63
336	367	336	0,64
337	370	337	0,66
337	468	337	0,67
337	443	337	0,67
337	367	337	0,67
338	330	338	0,67
338	329	338	0,69
340	305	340	0,69
340	278	340	0,69
340	276	340	0,69
340	332	340	0,69
342	389	342	0,7
342	303	342	0,7
342	305	342	0,7
344	383	344	0,71
344	359	344	0,72
346	368	346	0,72
346	279	346	0,73
349	270	349	0,74
349	268	349	0,74
349	383	349	0,75
350	391	350	0,75
353	576	353	0,75
353	596	353	0,75
355	367	355	0,75
355	379	355	0,75
355	397	355	0,75
355	357	355	0,75
356	569	356	0,76
356	542	356	0,76
357	348	357	0,76
357	290	357	0,76
357	234	357	0,76
357	239	357	0,77
359	248	359	0,77
360	292	360	0,77
360	400	360	0,78
361	372	361	0,78
361	228	361	0,78
363	195	363	0,78
365	604	365	0,78

367	596	367	0,78
367	382	367	0,79
367	364	367	0,8
367	314	367	0,8
368	279	368	0,81
368	323	368	0,81
369	319	369	0,81
369	289	369	0,82
371	257	371	0,82
371	695	371	0,82
371	616	371	0,82
372	376	372	0,82
372	376	372	0,82
372	924	372	0,82
373	733	373	0,83
373	279	373	0,83
375	258	375	0,83
375	249	375	0,84
375	248	375	0,84
377	320	377	0,84
377	304	377	0,84
377	282	377	0,84
379	303	379	0,84
379	358	379	0,85
380	356	380	0,85
380	729	380	0,85
380	494	380	0,85
380	445	380	0,85
381	434	381	0,85
381	350	381	0,86
383	426	383	0,86
384	246	384	0,86
384	750	384	0,86
384	416	384	0,86
386	213	386	0,86
386	436	386	0,87
387	279	387	0,87
387	776	387	0,87
387	380	387	0,87
387	313	387	0,87
388	290	388	0,87
388	426	388	0,88
388	311	388	0,88
390	358	390	0,88
390	483	390	0,88
390	347	390	0,88
390	256	390	0,88
390	839	390	0,88
391	317	391	0,89
391	278	391	0,89

392	442	392	0,89
392	234	392	0,89
392	234	392	0,89
394	445	394	0,89
394	206	394	0,89
395	327	395	0,9
395	338	395	0,9
396	309	396	0,91
396	255	396	0,91
396	357	396	0,91
396	474	396	0,91
398	756	398	0,91
398	281	398	0,91
398	331	398	0,91
398	617	398	0,91
399	631	399	0,91
399	400	399	0,91
400	384	400	0,91
400	354	400	0,91
400	373	400	0,91
402	387	402	0,91
402	269	402	0,91
402	430	402	0,91
402	495	402	0,91
402	248	402	0,91
403	464	403	0,91
404	291	404	0,92
404	692	404	0,92
404	391	404	0,92
404	501	404	0,92
404	623	404	0,92
404	411	404	0,92
404	319	404	0,92
404	441	404	0,92
404	649	404	0,92
406	256	406	0,92
407	337	407	0,92
407	537	407	0,93
407	606	407	0,93
407	387	407	0,93
408	365	408	0,93
408	397	408	0,93
410	262	410	0,94
410	280	410	0,94
411	317	411	0,94
411	480	411	0,94
411	356	411	0,94
412	148	412	0,94
412	519	412	0,94
412	430	412	0,94

414	503	414	0,94
414	261	414	0,94
415	261	415	0,94
415	265	415	0,94
415	275	415	0,94
417	279	417	0,94
418	284	418	0,94
418	284	418	0,95
419	285	419	0,95
419	285	419	0,95
419	287	419	0,95
421	292	421	0,95
421	298	421	0,95
422	303	422	0,95
422	305	422	0,95
422	306	422	0,95
422	306	422	0,95
422	306	422	0,95
423	310	423	0,95
423	314	423	0,96
423	314	423	0,96
423	315	423	0,96
423	315	423	0,96
425	315	425	0,96
425	316	425	0,96
426	319	426	0,96
426	319	426	0,96
427	319	427	0,96
427	324	427	0,96
427	324	427	0,96
427	325	427	0,96
429	325	429	0,96
429	327	429	0,96
429	327	429	0,96
430	329	430	0,97
430	331	430	0,97
431	331	431	0,97
431	331	431	0,97
431	332	431	0,97
431	336	431	0,97
431	338	431	0,97
431	338	431	0,97
433	340	433	0,97
433	340	433	0,97
433	340	433	0,97
433	340	433	0,97
433	340	433	0,97
433	341	433	0,97
434	341	434	0,97
435	343	435	0,97

435	343	435	0,97
435	343	435	0,98
437	343	437	0,98
437	345	437	0,98
438	345	438	0,98
438	349	438	0,98
438	349	438	0,98
438	349	438	0,99
438	350	438	0,99
439	350	439	0,99
441	350	441	0,99
441	351	441	0,99
441	351	441	0,99
441	351	441	0,99
442	354	442	0,99
442	354	442	0,99
442	355	442	0,99
442	355	442	0,99
443	355	443	0,99
445	356	445	0,99
445	356	445	0,99
445	356	445	0,99
445	359	445	0,99
446	359	446	1
446	360	446	1
448	360	448	1
448	360	448	1
449	360	449	1
449	360	449	1
449	362	449	1
449	362	449	1
450	362	450	1
450	362	450	1
450	365	450	1
450	367	450	1
450	367	450	1
450	369	450	1
450	371	450	1
452	371	452	1
452	371	452	1
452	371	452	1,01
452	372	452	1,01
453	372	453	1,01
453	372	453	1,01
453	374	453	1,01
454	374	454	1,01
454	374	454	1,01
454	374	454	1,01
454	374	454	1,01
457	376	457	1,01

457	376	457	1,01
460	376	460	1,01
461	376	461	1,01
461	376	461	1,01
462	378	462	1,01
462	378	462	1,01
462	380	462	1,01
462	380	462	1,01
464	380	464	1,01
464	381	464	1,02
464	381	464	1,02
465	384	465	1,02
465	384	465	1,02
465	384	465	1,02
466	384	466	1,02
466	384	466	1,02
466	385	466	1,02
466	385	466	1,02
468	385	468	1,02
468	385	468	1,02
469	385	469	1,02
469	385	469	1,02
470	385	470	1,02
472	386	472	1,02
472	386	472	1,02
472	386	472	1,02
473	386	473	1,02
474	386	474	1,03
474	386	474	1,03
474	386	474	1,03
474	389	474	1,03
474	389	474	1,03
474	389	474	1,03
476	390	476	1,03
476	390	476	1,03
480	390	480	1,03
480	390	480	1,03
481	390	481	1,03
481	390	481	1,03
481	391	481	1,03
483	391	483	1,03
484	391	484	1,03
484	394	484	1,03
484	394	484	1,03
485	394	485	1,03
485	394	485	1,03
487	394	487	1,03
487	394	487	1,03
487	395	487	1,04
487	395	487	1,04

487	395	487	1,04
487	396	487	1,04
488	396	488	1,04
489	396	489	1,04
491	396	491	1,04
492	396	492	1,04
492	396	492	1,04
493	396	493	1,04
495	396	495	1,04
495	399	495	1,04
495	399	495	1,04
496	399	496	1,04
496	399	496	1,04
497	399	497	1,04
497	399	497	1,04
499	399	499	1,04
499	400	499	1,05
499	400	499	1,05
501	400	501	1,05
503	400	503	1,05
504	400	504	1,05
508	400	508	1,05
508	402	508	1,05
508	402	508	1,05
512	404	512	1,05
514	404	514	1,05
515	404	515	1,05
515	404	515	1,05
516	405	516	1,05
516	405	516	1,05
519	405	519	1,05
519	405	519	1,05
520	405	520	1,05
522	407	522	1,05
523	407	523	1,05
527	407	527	1,05
527	407	527	1,05
528	412	528	1,05
531	412	531	1,05
531	412	531	1,05
532	412	532	1,05
534	414	534	1,06
535	414	535	1,06
535	414	535	1,06
537	416	537	1,06
538	416	538	1,06
538	416	538	1,06
541	416	541	1,06
542	416	542	1,06
542	418	542	1,06

543	418	543	1,06
546	418	546	1,06
546	418	546	1,06
549	418	549	1,06
549	418	549	1,06
559	418	559	1,06
562	418	562	1,06
563	420	563	1,06
565	420	565	1,06
566	420	566	1,06
568	420	568	1,06
568	421	568	1,06
568	421	568	1,06
569	424	569	1,06
570	424	570	1,06
572	424	572	1,06
573	424	573	1,07
580	425	580	1,07
585	425	585	1,07
585	425	585	1,07
588	426	588	1,07
589	426	589	1,07
590	426	590	1,07
597	429	597	1,07
603	429	603	1,07
604	429	604	1,07
605	430	605	1,07
613	430	613	1,07
616	430	616	1,07
619	431	619	1,07
620	431	620	1,07
627	431	627	1,08
627	431	627	1,08
628	431	628	1,08
628	434	628	1,08
632	434	632	1,08
639	434	639	1,08
640	434	640	1,08
644	434	644	1,08
654	435	654	1,08
655	435	655	1,08
663	435	663	1,08
667	435	667	1,08
674	436	674	1,08
764	436	764	1,08
787	436	787	1,08
851	436	851	1,08
900	439	900	1,08
906	439	906	1,08
956	439	956	1,08

2103	439	2103	1,09
	439	441	1,09
	440	383	1,09
	440	467	1,09
	440	377	1,09
	440	660	1,09
	440	623	1,09
	440	363	1,09
	442	381	1,09
	442	367	1,09
	444	370	1,09
	444	468	1,09
	444	443	1,09
	444	367	1,09
	445	330	1,09
	445	329	1,09
	445	305	1,09
	445	278	1,09
	445	276	1,09
	447	332	1,09
	447	389	1,09
	447	303	1,09
	447	305	1,09
	447	383	1,09
	447	359	1,09
	449	368	1,09
	449	279	1,09
	449	270	1,1
	449	268	1,1
	449	383	1,1
	449	391	1,1
	451	576	1,1
	451	596	1,1
	451	367	1,1
	451	379	1,1
	451	397	1,11
	452	357	1,11
	452	569	1,11
	452	542	1,11
	455	348	1,11
	455	290	1,11
	456	234	1,11
	456	239	1,11
	456	248	1,11
	456	292	1,11
	458	400	1,11
	458	372	1,11
	458	228	1,12
	458	195	1,12
	460	604	1,12

460	596	1,12
460	382	1,12
460	364	1,12
461	314	1,12
461	279	1,12
461	323	1,12
461	319	1,12
464	289	1,12
464	257	1,12
464	695	1,12
465	616	1,12
465	376	1,12
465	376	1,12
465	924	1,12
465	733	1,12
466	279	1,12
466	258	1,12
466	249	1,13
466	248	1,13
466	320	1,13
469	304	1,13
470	282	1,13
470	303	1,13
470	358	1,13
470	356	1,13
470	729	1,13
471	494	1,13
471	445	1,13
471	434	1,13
471	350	1,13
474	426	1,13
474	246	1,13
474	750	1,13
474	416	1,14
474	213	1,14
475	436	1,14
475	279	1,14
476	776	1,14
476	380	1,14
476	313	1,14
476	290	1,14
476	426	1,14
479	311	1,14
479	358	1,14
479	483	1,14
479	347	1,14
480	256	1,14
480	839	1,15
482	317	1,15
482	278	1,15

482	442	1,15
482	234	1,15
484	234	1,15
484	445	1,15
484	206	1,15
484	327	1,15
484	338	1,15
484	309	1,15
484	255	1,15
484	357	1,15
484	474	1,15
486	756	1,15
486	281	1,15
487	331	1,15
487	617	1,16
487	631	1,16
487	400	1,16
487	384	1,16
487	354	1,16
489	373	1,16
489	387	1,16
489	269	1,16
489	430	1,16
489	495	1,16
489	248	1,16
491	464	1,16
491	291	1,16
491	692	1,16
492	391	1,16
492	501	1,16
492	623	1,16
495	411	1,16
496	319	1,16
496	441	1,16
498	649	1,16
498	256	1,16
498	337	1,16
498	537	1,16
500	606	1,17
500	387	1,17
500	365	1,17
501	397	1,17
501	262	1,17
504	280	1,17
504	317	1,17
504	480	1,17
504	356	1,17
504	148	1,17
504	519	1,17
504	430	1,17

505	503	1,17
505	261	1,17
506	261	1,17
509	265	1,17
509	275	1,17
509	279	1,17
509	284	1,17
509	284	1,17
510	285	1,17
510	285	1,18
510	287	1,18
510	292	1,18
511	298	1,18
511	303	1,18
511	305	1,18
511	306	1,18
514	306	1,18
514	306	1,18
514	310	1,18
514	314	1,18
515	314	1,18
515	315	1,18
515	315	1,18
517	315	1,18
519	316	1,18
519	319	1,18
519	319	1,19
519	319	1,19
522	324	1,19
522	324	1,19
524	325	1,19
524	325	1,19
526	327	1,19
526	327	1,19
527	329	1,19
527	331	1,19
527	331	1,19
529	331	1,19
529	332	1,19
531	336	1,19
531	338	1,19
532	338	1,19
532	340	1,19
532	340	1,19
532	340	1,19
532	340	1,2
532	340	1,2
535	341	1,2
535	341	1,2
536	343	1,2

536	343	1,2
536	343	1,2
536	343	1,2
538	345	1,2
540	345	1,2
540	349	1,2
540	349	1,2
540	349	1,2
541	350	1,2
544	350	1,2
546	350	1,2
546	351	1,2
549	351	1,2
549	351	1,2
550	354	1,2
550	354	1,2
550	355	1,21
550	355	1,21
551	355	1,21
551	356	1,21
554	356	1,21
554	356	1,21
554	359	1,21
555	359	1,21
555	360	1,21
557	360	1,21
557	360	1,21
559	360	1,21
559	360	1,21
560	362	1,21
560	362	1,21
564	362	1,21
567	362	1,21
567	365	1,21
569	367	1,21
571	367	1,21
575	369	1,21
575	371	1,21
575	371	1,22
576	371	1,22
576	371	1,22
578	372	1,22
578	372	1,22
580	372	1,22
580	374	1,22
581	374	1,22
581	374	1,22
584	374	1,22
585	374	1,22
585	376	1,22

589	376	1,22
590	376	1,22
590	376	1,22
591	376	1,22
591	378	1,23
591	378	1,23
595	380	1,23
597	380	1,23
597	380	1,23
597	381	1,23
597	381	1,23
597	384	1,23
602	384	1,23
602	384	1,23
607	384	1,24
611	384	1,24
615	385	1,24
615	385	1,24
615	385	1,24
616	385	1,24
621	385	1,24
624	385	1,24
625	385	1,24
629	386	1,24
629	386	1,24
630	386	1,24
630	386	1,24
630	386	1,24
630	386	1,24
644	386	1,25
644	389	1,25
646	389	1,25
659	389	1,25
659	390	1,25
660	390	1,25
661	390	1,25
665	390	1,25
669	390	1,25
670	390	1,25
670	391	1,25
671	391	1,25
681	391	1,25
681	394	1,25
684	394	1,25
691	394	1,25
699	394	1,25
701	394	1,25
704	394	1,25
706	395	1,25
706	395	1,25

714	395	1,25
714	396	1,25
715	396	1,25
726	396	1,26
745	396	1,26
755	396	1,26
757	396	1,26
770	396	1,26
773	396	1,26
792	399	1,26
820	399	1,26
837	399	1,26
865	399	1,26
877	399	1,26
909	399	1,26
912	399	1,26
956	400	1,27
971	400	1,27
975	400	1,27
992	400	1,27
992	400	1,27
1007	400	1,27
1007	402	1,27
1056	402	1,27
1110	404	1,27
2030	404	1,27
2367	404	1,27
	404	1,27
	405	1,27
	405	1,27
	405	1,27
	405	1,27
	405	1,27
	407	1,27
	407	1,27
	407	1,27
	407	1,28
	412	1,28
	412	1,28
	412	1,28
	412	1,28
	414	1,28
	414	1,28
	414	1,28
	416	1,28
	416	1,29
	416	1,29
	416	1,29
	416	1,29
	418	1,29

418	1,29
418	1,29
418	1,29
418	1,29
418	1,29
418	1,29
418	1,29
420	1,3
420	1,3
420	1,3
420	1,3
421	1,3
421	1,3
424	1,3
424	1,3
424	1,3
424	1,3
425	1,3
425	1,3
425	1,3
426	1,3
426	1,3
426	1,3
429	1,3
429	1,3
429	1,3
430	1,3
430	1,31
430	1,31
431	1,31
431	1,31
431	1,31
431	1,31
431	1,31
434	1,31
434	1,31
434	1,31
434	1,31
434	1,31
435	1,31
435	1,31
435	1,31
435	1,31
436	1,31
436	1,31
436	1,31
436	1,31
439	1,31
439	1,31
439	1,32

439	1,32
439	1,32
440	1,32
440	1,32
440	1,32
440	1,32
440	1,32
440	1,32
442	1,33
442	1,33
444	1,33
444	1,33
444	1,33
444	1,33
445	1,33
445	1,33
445	1,33
445	1,33
445	1,33
447	1,34
447	1,34
447	1,34
447	1,34
447	1,34
447	1,34
449	1,34
449	1,34
449	1,34
449	1,34
449	1,34
449	1,34
451	1,35
451	1,35
451	1,35
451	1,35
451	1,35
452	1,35
452	1,35
452	1,35
455	1,35
455	1,35
456	1,35
456	1,35
456	1,35
456	1,35
458	1,36
458	1,36
458	1,36
458	1,37
460	1,37

460	1,37
460	1,37
460	1,37
461	1,37
461	1,37
461	1,37
461	1,37
464	1,37
464	1,37
464	1,37
465	1,37
465	1,37
465	1,37
465	1,37
465	1,38
466	1,38
466	1,38
466	1,38
466	1,38
466	1,38
469	1,38
470	1,38
470	1,38
470	1,38
470	1,38
470	1,39
471	1,39
471	1,39
471	1,4
471	1,4
474	1,4
474	1,4
474	1,4
474	1,4
474	1,4
475	1,4
475	1,4
476	1,4
476	1,4
476	1,41
476	1,41
476	1,41
479	1,41
479	1,41
479	1,42
479	1,42
480	1,42
480	1,42
482	1,42
482	1,42

482	1,42
482	1,42
484	1,43
484	1,43
484	1,43
484	1,43
484	1,43
484	1,43
484	1,43
484	1,43
484	1,43
486	1,43
486	1,43
487	1,44
487	1,44
487	1,44
487	1,44
487	1,44
487	1,44
489	1,44
489	1,44
489	1,44
489	1,44
489	1,44
489	1,45
491	1,45
491	1,45
491	1,45
492	1,45
492	1,45
492	1,45
495	1,45
496	1,45
496	1,46
498	1,46
498	1,46
498	1,46
498	1,46
500	1,47
500	1,47
500	1,47
501	1,47
501	1,48
504	1,48
504	1,48
504	1,48
504	1,48
504	1,48
504	1,48
504	1,48

505	1,48
505	1,48
506	1,49
509	1,49
509	1,49
509	1,49
509	1,49
509	1,5
510	1,5
510	1,5
510	1,5
510	1,5
511	1,51
511	1,51
511	1,51
511	1,51
514	1,52
514	1,52
514	1,52
514	1,52
515	1,52
515	1,53
515	1,53
517	1,53
519	1,53
519	1,53
519	1,53
519	1,53
522	1,53
522	1,54
524	1,54
524	1,55
526	1,55
526	1,55
527	1,55
527	1,55
527	1,55
529	1,55
529	1,55
531	1,56
531	1,56
532	1,56
532	1,56
532	1,56
532	1,57
532	1,57
532	1,57
535	1,57
535	1,57
536	1,58

536	1,58
536	1,58
536	1,59
538	1,59
540	1,59
540	1,59
540	1,59
540	1,59
541	1,6
544	1,6
546	1,6
546	1,6
549	1,6
549	1,6
550	1,6
550	1,6
550	1,6
550	1,62
551	1,62
551	1,62
554	1,62
554	1,62
554	1,63
555	1,63
555	1,63
557	1,64
557	1,65
559	1,65
559	1,65
560	1,65
560	1,66
564	1,66
567	1,66
567	1,66
569	1,66
571	1,67
575	1,67
575	1,67
575	1,67
576	1,68
576	1,68
578	1,69
578	1,69
580	1,69
580	1,69
581	1,69
581	1,69
584	1,69
585	1,69
585	1,69

589	1,69
590	1,7
590	1,7
591	1,72
591	1,72
591	1,73
595	1,73
597	1,73
597	1,74
597	1,74
597	1,76
597	1,76
602	1,77
602	1,77
607	1,77
611	1,77
615	1,78
615	1,78
615	1,79
616	1,79
621	1,8
624	1,8
625	1,8
629	1,8
629	1,81
630	1,83
630	1,83
630	1,84
630	1,86
644	1,86
644	1,87
646	1,88
659	1,88
659	1,89
660	1,9
661	1,9
665	1,92
669	1,92
670	1,92
670	1,95
671	1,96
681	1,97
681	2
684	2,02
691	2,03
699	2,03
701	2,03
704	2,05
706	2,07
706	2,08

714	2,09
714	2,12
715	2,13
726	2,2
745	2,25
755	2,26
757	2,29
770	2,33
773	2,36
792	2,42
820	2,44
837	2,44
865	2,45
877	2,48
909	2,57
912	2,57
956	2,61
971	2,62
975	2,67
992	2,67
992	2,71
1007	2,71
1007	2,84
1056	2,98
1110	5,46
2030	5,65
2367	6,36

controls	PCOS-A	PCOS-HU	PCOS-DE
283	260,84	441,11	281,98
295	260,84	382,74	302,56
299	264,71	467,48	358,23
307	275,04	377,09	355,81
311	278,92	660,49	728,56
325	284,08	623,3	494,38
326	284,08	362,96	445,37
328	285,37	380,85	434,47
336	285,37	367,2	349,76
337	286,66	370,03	426
337	291,83	468,42	246,28
337	298,28	443,47	749,74
337	303,45	367,2	415,72
338	304,74	330,01	213
338	306,03	328,6	436,29
340	306,03	305,06	278,96
340	306,03	277,76	775,76
340	309,91	275,87	380,01
340	313,78	332,36	313,45
342	313,78	388,86	290,46
342	315,07	302,71	426
342	315,07	304,59	311,03
344	315,07	383,21	358,23
344	316,36	359,2	482,88
346	318,94	368,14	347,34
346	318,94	278,7	255,96
349	318,94	269,75	838,69
349	324,11	267,87	317,08
349	324,11	383,21	278,35
350	325,4	390,74	442,34
353	325,4	576,22	233,58
353	326,69	596	233,58
355	326,69	366,73	445,37
355	329,27	378,97	205,74
355	330,57	397,33	327,37
355	330,57	357,32	337,66
356	330,57	569,16	309,22
356	331,86	541,86	255,36
357	335,73	348,37	357,02
357	338,31	289,52	473,81
357	338,31	233,5	756,4
357	339,61	238,68	280,77
359	339,61	247,63	331
360	339,61	291,88	616,61
360	339,61	399,68	630,53
361	339,61	372,38	399,98
361	340,9	228,32	384,25
363	340,9	195,37	353,99
365	343,48	604	372,75

367	343,48	596,47	386,67
367	343,48	381,8	269,28
367	343,48	364,38	429,63
367	344,77	314	494,99
368	344,77	279,17	248,1
368	348,64	322,95	464,13
369	348,64	319,18	291,06
369	348,64	288,58	691,65
371	349,94	256,57	390,91
371	349,94	695,33	501,04
371	349,94	615,77	622,67
372	351,23	375,68	410,87
372	351,23	375,68	318,9
372	351,23	924,12	440,53
373	353,81	732,52	648,69
373	353,81	279,17	255,96
375	355,1	258,45	337,05
375	355,1	248,57	537,34
375	355,1	247,63	606,33
377	356,39	319,65	387,28
377	356,39	304,12	365,49
377	356,39		396,96
379	358,97		262,02
379	358,97		279,56
380	360,27		317,08
380	360,27		480,46
380	360,27		356,41
380	360,27		148,25
381	360,27		518,59
381	361,56		429,63
383	361,56		503,46
384	361,56		
384	361,56		
384	365,43		
386	366,72		
386	366,72		
387	369,3		
387	370,6		
387	370,6		
387	370,6		
388	370,6		
388	371,89		
388	371,89		
390	371,89		
390	374,47		
390	374,47		
390	374,47		
390	374,47		
391	374,47		
391	375,76		

392	375,76
392	375,76
392	375,76
394	375,76
394	378,34
395	378,34
395	379,63
396	379,63
396	379,63
396	380,93
396	380,93
398	383,51
398	383,51
398	383,51
398	383,51
399	383,51
399	384,8
400	384,8
400	384,8
400	384,8
402	384,8
402	384,8
402	384,8
402	386,09
402	386,09
403	386,09
404	386,09
404	386,09
404	386,09
404	386,09
404	388,67
404	388,67
404	388,67
404	389,96
404	389,96
406	389,96
407	389,96
407	389,96
407	389,96
407	391,26
408	391,26
408	391,26
410	393,84
410	393,84
411	393,84
411	393,84
411	393,84
412	393,84
412	395,13
412	395,13

414	395,13
414	396,42
415	396,42
415	396,42
415	396,42
417	396,42
418	396,42
418	396,42
419	396,42
419	399
419	399
421	399
421	399
422	399
422	399
422	399
422	400,29
422	400,29
423	400,29
423	400,29
423	400,29
423	400,29
423	401,59
425	401,59
425	404,17
426	404,17
426	404,17
427	404,17
427	405,46
427	405,46
427	405,46
429	405,46
429	405,46
429	406,75
430	406,75
430	406,75
431	406,75
431	411,92
431	411,92
431	411,92
431	411,92
431	414,5
433	414,5
433	414,5
433	415,79
433	415,79
433	415,79
433	415,79
434	415,79
435	418,37

435	418,37
435	418,37
437	418,37
437	418,37
438	418,37
438	418,37
438	418,37
438	419,66
438	419,66
439	419,66
441	419,66
441	420,96
441	420,96
441	423,54
442	423,54
442	423,54
442	423,54
442	424,83
443	424,83
445	424,83
445	426,12
445	426,12
445	426,12
446	428,7
446	428,7
448	428,7
448	429,99
449	429,99
449	429,99
449	431,29
449	431,29
450	431,29
450	431,29
450	431,29
450	433,87
450	433,87
450	433,87
450	433,87
452	433,87
452	435,16
452	435,16
452	435,16
453	435,16
453	436,45
453	436,45
454	436,45
454	436,45
454	439,03
454	439,03
457	439,03

457	439,03
460	439,03
461	440,32
461	440,32
462	440,32
462	440,32
462	440,32
462	440,32
464	441,62
464	441,62
464	444,2
465	444,2
465	444,2
465	444,2
466	445,49
466	445,49
466	445,49
466	445,49
468	445,49
468	446,78
469	446,78
469	446,78
470	446,78
472	446,78
472	446,78
472	449,36
473	449,36
474	449,36
474	449,36
474	449,36
474	449,36
474	450,65
474	450,65
476	450,65
476	450,65
480	450,65
480	451,95
481	451,95
481	451,95
481	454,53
483	454,53
484	455,82
484	455,82
484	455,82
485	455,82
485	458,4
487	458,4
487	458,4
487	458,4
487	459,69

487	459,69
487	459,69
488	459,69
489	460,98
491	460,98
492	460,98
492	460,98
493	463,57
495	463,57
495	463,57
495	464,86
496	464,86
496	464,86
497	464,86
497	464,86
499	466,15
499	466,15
499	466,15
501	466,15
503	466,15
504	468,73
508	470,02
508	470,02
508	470,02
512	470,02
514	470,02
515	471,31
515	471,31
516	471,31
516	471,31
519	473,9
519	473,9
520	473,9
522	473,9
523	473,9
527	475,19
527	475,19
528	476,48
531	476,48
531	476,48
532	476,48
534	476,48
535	479,06
535	479,06
537	479,06
538	479,06
538	480,35
541	480,35
542	481,65
542	481,65

543	481,65
546	481,65
546	484,23
549	484,23
549	484,23
559	484,23
562	484,23
563	484,23
565	484,23
566	484,23
568	484,23
568	485,52
568	485,52
569	486,81
570	486,81
572	486,81
573	486,81
580	486,81
585	486,81
585	489,39
588	489,39
589	489,39
590	489,39
597	489,39
603	489,39
604	490,68
605	490,68
613	490,68
616	491,98
619	491,98
620	491,98
627	494,56
627	495,85
628	495,85
628	498,43
632	498,43
639	498,43
640	498,43
644	499,72
654	499,72
655	499,72
663	501,01
667	501,01
674	503,6
764	503,6
787	503,6
851	503,6
900	503,6
906	503,6
956	503,6

2103	504,89
	504,89
	506,18
	508,76
	508,76
	508,76
	508,76
	508,76
	510,05
	510,05
	510,05
	510,05
	511,34
	511,34
	511,34
	511,34
	513,93
	513,93
	513,93
	513,93
	515,22
	515,22
	515,22
	516,51
	519,09
	519,09
	519,09
	519,09
	521,67
	521,67
	524,26
	524,26
	525,55
	525,55
	526,84
	526,84
	526,84
	529,42
	529,42
	530,71
	530,71
	532
	532
	532
	532
	532
	532
	534,59
	534,59
	535,88

535,88
535,88
535,88
538,46
539,75
539,75
539,75
539,75
541,04
543,63
546,21
546,21
548,79
548,79
550,08
550,08
550,08
550,08
551,37
551,37
553,96
553,96
553,96
555,25
555,25
556,54
556,54
559,12
559,12
560,41
560,41
564,29
566,87
566,87
569,45
570,74
574,62
574,62
574,62
575,91
575,91
578,49
578,49
579,78
579,78
581,07
581,07
583,66
584,95
584,95

588,82
590,11
590,11
591,4
591,4
591,4
595,28
596,57
596,57
596,57
596,57
596,57
601,73
601,73
606,9
610,77
614,65
614,65
614,65
615,94
621,1
623,69
624,98
628,85
628,85
630,14
630,14
630,14
630,14
644,35
644,35
645,64
658,55
658,55
659,84
661,13
665,01
668,88
670,17
670,17
671,46
680,5
680,5
684,38
690,83
698,58
701,16
703,74
706,33
706,33

714,07
714,07
715,37
725,7
745,07
755,4
756,69
769,6
773,47
791,55
819,96
836,75
865,15
876,77
909,06
911,64
955,54
971,04
974,91
991,7
991,7
1007,19
1007,19
1056,26
1110,5
2029,88
2366,9

control		positive		equal vol. mix	
551	632	2382	2151	1460	1448
656	714	1903	1744	1287	1221
604	640	1952	1949	1192	1263

total Testost. [ng/ml]	free Testost. [pg/ml]	DHEAS [µg/ml]
0,01	1,41	1,06
0,02	1,89	1,48
0,09	2,83	1,34
0,11	1,65	1,74
0,12	2,56	1,07
0,12	1,15	0,63
0,13	1,27	1,01
0,13	1,71	0,79
0,14	5,78	3,03
0,14	1,5	0,83
0,17	1,81	2,75
0,17	1,23	1,14
0,17	1,35	0,44
0,17	1,32	2,08
0,17	1,92	1,47
0,18	2,56	2,7
0,18	1,38	0,63
0,19	1,82	1,67
0,19	2,87	2,04
0,2	1,19	1,04
0,2	2,01	1,2
0,2	3,5	2,92
0,2	2,29	2,7
0,21	1,07	1,16
0,21	1,04	0,95
0,21	1,87	1,19
0,22	1,42	0,49
0,22	1,59	1,36
0,22	2,09	1,86
0,22	1,34	0,99
0,22	2,44	
0,22	1,49	0,97
0,24	1,82	0,54
0,24	1,83	0,57
0,24	1,7	1,15
0,24	2,97	1,28
0,24	1,18	1,81
0,24	2,57	2,61
0,24	4,03	2,43
0,24	2,13	1,52
0,24	2,96	0,76
0,25	2,74	0,68
0,25	1,53	1,33
0,25	1,43	1,4
0,25	1,66	1,28
0,25	1,4	1,16
0,26	2,6	4,38
0,26	1,93	1,97
0,26	0,8	2,82

0,28	1,71	1,3
0,28	2,26	3,55
0,28	1,97	2,07
0,29	1,68	2,14
0,29	1,15	2,2
0,29	2,62	1,76
0,29	1,7	1
0,3	2,31	2,4
0,3	1,86	1,06
0,3	2,19	2,54
0,3	0,99	0,87
0,3	1,31	1,56
0,3	2,5	4,75
0,31	1,56	1,08
0,31	1,82	2,15
0,31	3,59	4,8
0,31	1,53	1,15
0,32	0,68	0,78
0,32	2,76	1,98
0,32	3,33	2,46
0,32	1,22	0,86
0,32	2,34	2,43
0,33	1,93	1,29
0,33	2,06	0,88
0,33	1,11	1,19
0,33	2,58	2,52
0,33	1,29	1,13
0,33	2,03	1,87
0,34	2,69	3,4
0,34	1,39	1,97
0,34	1,58	3,13
0,35	1,91	2,06
0,35	2,71	2
0,35	1,73	2,48
0,35	1,13	0,97
0,35	2,42	5,27
0,35	1,89	2,81
0,35	1,43	1,11
0,35	2,4	2,79
0,35	1,44	2,29
0,35	1,62	1,22
0,35	1,1	1,17
0,36	1,75	1,83
0,36	1,35	1,25
0,36	3,1	1,96
0,36	1,98	1,59
0,36	1,58	1,43
0,36	1,65	2,58
0,36	1,66	3,47
0,37	3,01	2,76

0,37	1,22	2,3
0,37	1,58	0,86
0,37	2,11	1,2
0,37	1,88	1,72
0,37	2,78	2,87
0,37	2,03	1,74
0,37	2,63	2,8
0,37	2,93	3,51
0,37	1,89	1,97
0,38	2,05	1,43
0,38	1,98	2,52
0,38	1,57	1,1
0,38	1,28	0,65
0,38	2,65	2,16
0,38	1,76	1,1
0,38	2,23	1,51
0,39	1,55	1,97
0,39	1,71	1
0,39	1,46	4,16
0,39	1,65	6,51
0,39	3,5	2,02
0,39	5,39	2,28
0,39	5,01	1,67
0,4	2,05	2,12
0,4	2,89	2,86
0,4	1,48	3
0,4	1,99	3,11
0,4	2,44	1,47
0,4	2,42	2,92
0,4	1,64	1,07
0,4	1,65	1,02
0,4	3,27	2,87
0,4	1,54	1,5
0,41	2,59	2,05
0,41	2,76	0,73
0,41	3,63	2,59
0,41	2,26	1,77
0,41	2,48	0,37
0,41	2,44	2,15
0,41	1,71	2,11
0,41	2,15	2,44
0,41	1,66	5,6
0,41	0,97	1,31
0,42	1,77	3,04
0,42	3,19	1,72
0,42	4,24	2,92
0,42	2,16	2,53
0,42	1,65	1,23
0,42	1,53	0,92
0,43	1,5	2,25

0,43	1,85	2,5
0,43	0,9	1,08
0,44	3,3	0,5
0,44	2,51	0,78
0,44	2,68	3,4
0,44	2,12	2,48
0,44	4,38	2,97
0,44	3,15	1,17
0,44	2,29	1,78
0,44	2,97	1,99
0,45	0,92	0,7
0,45	3,43	2,65
0,45	2,06	1,84
0,45	2,13	2,4
0,45	1,06	0,86
0,46	1,19	1,7
0,46	1,61	0,7
0,46	2,2	1,89
0,46	3,69	2,32
0,46	3,69	5,97
0,46	2,07	1,19
0,46	2,72	1,62
0,46	2,11	1,29
0,47	2,17	1,47
0,47	1,97	2,07
0,47	1,3	1,12
0,47	2,29	2,54
0,47	1,98	2,41
0,47	1,72	1,16
0,47	2,97	1,77
0,47	3,34	2,63
0,47	2,63	1,69
0,48	3,64	2,92
0,48	2,88	3,57
0,48	3,17	4,19
0,48	2,87	4,5
0,48	1,89	1,48
0,48	2,05	1,34
0,48	1,99	0,83
0,48	1,72	2,4
0,49	2,76	2,9
0,49	1,95	2,07
0,49	1,46	1,4
0,49	1,87	1,86
0,49	1,45	0,99
0,49	0,83	0,56
0,49	1,76	2,04
0,49	2,57	1,64
0,49	4,34	1,36
0,49	1,49	1,67

0,49	3,68	2,95
0,49	3,24	3,11
0,49	2,53	3,53
0,49	2,94	2,58
0,49	2,9	2,07
0,49	1,02	2,15
0,49	3,37	2,48
0,5	1,31	1,27
0,5	1,07	0,31
0,5	2,68	1,24
0,5	1,76	
0,5	2,39	3,03
0,5	2,63	2,89
0,5	1,36	1,79
0,5	3,25	1,76
0,5	2,87	2,3
0,5	1,44	0,92
0,5	2,04	1,12
0,5	3,58	2,42
0,51	2,56	3,48
0,51	3,58	3,21
0,51	2,11	1,49
0,51	1,42	1,06
0,51	2,32	3,07
0,51	3,52	1,34
0,51	2,18	1,43
0,51	1,97	2,8
0,51	2,22	2,66
0,51	2,28	4,43
0,51	2,19	1,45
0,51	1,92	0,96
0,52	2,34	2,71
0,52	4,4	0,87
0,52	3,37	2,39
0,52	1,46	1,6
0,52	4,42	2,13
0,52	3,23	2,08
0,53	5,03	1,55
0,53	2,2	2,87
0,53	2,29	4,21
0,53	1,95	0,7
0,53	3,54	3,03
0,53	2,23	2,36
0,53	2,04	1,74
0,53	2,48	1,78
0,53	5,41	2,17
0,53	3,72	2,45
0,54	4,28	3,37
0,54	3,19	2,24
0,54	1,43	0,92

0,54	1,33	0,54
0,54	2,53	1,44
0,54	3,71	3,87
0,55	3,38	2,43
0,55	3,51	1,28
0,55	3,51	1,85
0,55	3,29	4,4
0,56	1,88	3,52
0,56	1,71	0,11
0,56	2,46	1,47
0,56	1,54	1,7
0,56	3,27	2,87
0,56	2,14	1,55
0,56	3,86	1,85
0,57	1,51	1,3
0,57	2,71	1,75
0,57	1,78	1,5
0,57	1,1	1,11
0,57	4,72	5,14
0,58	3,77	1,65
0,58	6,04	4,01
0,58	3,55	2,55
0,58	3,43	3,26
0,58	1,84	1,47
0,59	4,21	4,52
0,59	3,6	2,76
0,59	3,51	
0,59	1,32	1,08
0,59	3,73	4,61
0,59	2,87	4,47
0,59	1,72	1,88
0,59	6,16	2,9
0,6	2,47	1,21
0,6	2,19	2,31
0,6	1,91	2
0,6	2,24	2,21
0,6	4,07	1,81
0,6	1,44	1,66
0,6	1,78	2,16
0,6	3,32	1,5
0,6	2,68	1,39
0,61	2,14	1,34
0,61	3,42	4,6
0,61	1,79	1,84
0,62	2,05	1,25
0,62	3,06	3,01
0,62	3,15	2,14
0,62	2,05	1,56
0,62	2,72	1,47
0,62	3,03	1,66

0,62	1,4	1,41
0,62	5,06	2,96
0,63	2,02	0,96
0,63	2,65	2,71
0,63	1,9	2,7
0,63	2,28	1,75
0,63	3,59	3,23
0,63	3,2	2,05
0,63	2,66	0,96
0,63	4,15	2,33
0,63	2,38	4,34
0,63	2,31	1,25
0,64	1,95	0,88
0,64	2,2	2,26
0,64	3,76	1,4
0,64	1,84	0,91
0,64	2,84	3,3
0,64	2,89	1,63
0,64	3,59	2,46
0,64	5,63	3,55
0,64	3,1	0,76
0,64	0,9	1,08
0,65	1,93	1,93
0,65	1,64	2,04
0,65	3,82	1,44
0,65	2,2	1,01
0,65	4,05	1,45
0,65	3,13	2,78
0,65	2,77	2,13
0,65	2	2,37
0,65	3,47	6,94
0,65	3,28	1,05
0,66	2,54	2,09
0,66	2,99	2,06
0,66	2,17	1,49
0,66	2,37	1,8
0,66	1,94	1,08
0,66	2,23	2,67
0,66	4,13	4,1
0,66	1,16	1,46
0,66	3,32	1,81
0,66	6,01	3,72
0,66	3,11	3,92
0,67	4,81	2,32
0,67	3	1,46
0,67	2,21	1,65
0,67	3,49	2,74
0,67	2,65	3,23
0,67	3,1	1,94
0,67	1,61	1,97

0,67	3,82	2,04
0,67	3,68	3,66
0,68	3,34	1,72
0,68	2,03	1,93
0,68	2,08	1,68
0,68	6,23	3,08
0,69	2,99	1,7
0,69	2,33	1,32
0,69	4,19	2,13
0,69	1,82	1,19
0,69	2,26	1,98
0,69	3,23	2,44
0,7	3,36	2,9
0,7	2,16	5,25
0,7	2,86	2,98
0,7	3,11	1,77
0,7	1,72	1,26
0,7	4,17	1,51
0,71	4,01	2,93
0,71	3,56	1,32
0,71	3,46	3,9
0,71	1,75	1,03
0,72	2,71	3,66
0,72	1,87	1,41
0,72	3,2	2,1
0,72	1,04	1,32
0,72	4,77	3,29
0,73	2,06	2,6
0,73	2,52	0,78
0,73	3,26	0,9
0,73	1,46	1,87
0,73	2,34	2,79
0,73	3,92	1,49
0,73	2,17	1,44
0,73	1,94	1,77
0,74	4,02	2,7
0,75	2,31	0,66
0,75	3,73	1,77
0,75	3,53	0,5
0,75	4,16	3,55
0,75	3,29	2,7
0,75	2,75	2,1
0,75	4,52	4,6
0,75	3,52	2,41
0,75	3,17	2,8
0,75	2,6	2,23
0,76	3,15	0,92
0,76	4,92	2,07
0,76	2,61	2,64
0,76	4,71	2,83

0,76	2,52	1,73
0,76	5,13	2,5
0,76	2,1	3,08
0,76	3,86	4,59
0,76	2,83	0,93
0,76	4,01	3,51
0,76	2,22	1,54
0,77	4,02	2,27
0,77	4,5	3
0,77	1,75	1,06
0,77	2,52	2,17
0,77	7,7	1,91
0,78	3,41	1,72
0,78	1,92	1,65
0,78	2,88	3,84
0,78	3,09	2,48
0,78	4,41	4,33
0,79	2,87	1,95
0,79	4,45	3,32
0,79	4,62	4,4
0,79	3,1	1,27
0,79	2,6	2,07
0,79	4,28	1,56
0,79	3,76	1,72
0,79	2,96	1,52
0,8	2,97	4,73
0,8	2,92	3,16
0,8	1,9	1,9
0,8	5,49	5,2
0,8	2,43	1,04
0,8	3,08	3,19
0,8	6,23	4,01
0,8	2,55	3,78
0,81	3,88	3,17
0,81	3,33	2,46
0,81	2,38	0,92
0,82	4,26	0,98
0,82	3,75	1,8
0,82	2,93	4,3
0,82	1,93	1,04
0,82	2,84	3,5
0,82	3,47	1,34
0,82	3,99	3,85
0,82	2,05	1,38
0,82	4,82	2,88
0,82	2,3	1,39
0,82	2,96	1,69
0,83	2,59	1,2
0,83	2,21	2,64
0,83	2,44	1,97

0,83	2,96	1,98
0,83	3,36	3,01
0,83	3,44	2,13
0,83	6,44	3,66
0,83	5,23	2,85
0,84	4,08	1,56
0,84	3,51	3,63
0,84	2,26	3
0,84	2,99	1,51
0,84	1,52	0,74
0,85	2,94	1,11
0,85	3,76	0,51
0,85	3,76	2,88
0,85	2,25	2,6
0,85	4,87	2,07
0,85	3,33	3,39
0,87	3,49	2,18
0,87	2,54	2,62
0,87	1,93	2,63
0,87	3,93	2,22
0,88	3,23	2,22
0,88	5,26	0,9
0,88	3,37	2,5
0,88	5,08	2,48
0,88	2,05	1,6
0,88	3,75	2,34
0,89	2,75	2,39
0,89	4,68	1,18
0,89	2,95	4,05
0,89	7,43	2,95
0,9	4,17	1,8
0,9	3,46	5,67
0,9	2,47	1,02
0,9	2,39	1,5
0,91	4,58	1,48
0,91	4,78	1,22
0,92	4,54	2,8
0,93	2,8	1,18
0,93	4,59	5,14
0,93	2,96	3,17
0,93	5,15	2,29
0,93	3,27	2,34
0,94	4,02	3,3
0,94	3,35	1,6
0,95	4,85	5,3
0,95	2,9	1,96
0,95	2,2	1,83
0,95	3,11	1,77
0,96	2,29	2,28
0,97	2,83	2,25

0,97	3,38	3,6
0,97	5,05	
0,97	3,06	1
0,98	2,31	2,6
0,98	2,31	1,47
0,98	4,19	2,62
0,99	4,95	3,07
1	4,25	1,94
1,01	4,23	3,91
1,01	3,95	1,21
1,02	2,88	2,12
1,02	1,88	1,41
1,03	3,51	1,91
1,04	2,94	1,66
1,05	3,68	2,58
1,06	3,29	3,13
1,06	2,92	3,35
1,06	2,48	1,42
1,06	2,08	2,1
1,07	3,56	2,8
1,08	3,95	4,11
1,09	3,5	1,55
1,09	6,88	2,92
1,09	6,51	5,16
1,1	3,91	2,57
1,1	3,58	8
1,11	6,77	2,56
1,12	2,68	1,43
1,13	1,02	0,75
1,13	3,22	3,24
1,14	3,82	2,8
1,15	5,73	2,11
1,16	3,4	3,16
1,16	4,55	2,45
1,16	2,27	2,13
1,17	5,64	2,72
1,22	7,23	7,23
1,27	7,39	4,09
1,29	3,06	0,92
1,31	4,25	2,08
1,32	6,96	1,79
1,35	2,14	1,8
1,37	5,71	4,2
1,39	5,97	1,44
1,4	4,67	2,1
1,43	3,51	1,9
1,44	5,22	3,5
1,5	3,99	2,26
1,77	1,56	1,98
1,79	3,99	3,83

2,57	2,13	1,77
	2,93	0,24
	1,14	1,35
	1,31	1,81
	2,47	2,83
	3,33	1,74

Androstenedione [ng/ml]
1,1
1,7
1,6
2,9
2,2
1,5
3
2,5
7
1,4
4,8
1,8
1,7
2,5
2,8
1,3
1,9
1,5
3
2,9
2,7
5
3,4
1,2
2,2
2,4
1,1
2
3,7
1,5
3,6
1,8
1,1
3,4
3,5
2,2
1,8
2,6
2,2
2,7
2,1
2,7
1,8
1,7
3,3
2,3
1,8
3,6
4,5

1,6
2,2
3,9
1,8
1,1
3,7
2
1,9
1,4
2,2
2
2
5,2
3,9
3,1
4,5
2,2
1
9,8
5
3
2,9
2,2
2,3
5
4
2,5
4,8
2,8
2
3,7
2
1,3
6,5
1,7
3,8
3,5
2,6
3,5
3,5
2,1
1,6
4
2,1
3,3
4,2
3,6
3,3
4,3
3,2

1,8
1,1
1,9
3,6
3,8
2,6
2,1
3,4
3,5
3,5
11,2
1,3
1,9
3,9
2,9
3,3
1,1
1,2
3,2
10
3,6
4,5
4,6
2,5
3,9
1,7
2,5
2,1
3,5
2,9
2,7
5,2
3,6
3
4,7
1,8
2,2
3,1
3,5
3,2
3,3
9
2,1
3,9
2,4
3,8
2,3
3,6
2,6
2,7

9,8
1,3
3,1
1,9
1,7
2,3
4,2
3,4
4,3
3,6
0,9
2,9
2,5
3,4
2,8
3
2,2
6,9
3,8
5,6
2,5
3,8
3
4,1
6,8
1,8
1,2
2,6
1,7
3,3
3,7
4,4
2,6
10
3,5
10
2,1
3,8
2,3
4,6
3,8
3,3
1,3
6,1
7,8
2
2,9
3,1
3,1
2,8

3,5
2,8
4,3
4,1
2,9
2,2
3,7
3
1,3
2,5
1,3
2,1
5,9
4
5,8
5,1
1,6
2,5
2,6
4,9
10
1,7
2,7
4,2
2,5
3,4
2,9
3,5
5
3,1
4,3
1,9
1,2
4,9
2,3
5
3,2
1,6
5,6
5,6
5,4
4,6
4,4
2,1
3,2
3,9
3,2
3,1
1,8
1,9

1,8
3,1
4,5
2,7
2,2
4,3
7,6
2,2
2
1,8
3,8
5,1
2,9
2,1
2,1
7
4,9
4
4,7
3,9
5,2
8,3
9,2
3,1
22,1
2,3
1,4
2
10
8,8
3,5
5,4
2,1
3,2
3,9
1,9
5,4
2,3
4,1
3,7
1,9
2
5,4
2,8
4,5
4,1
2,4
2,8
3,8
2,7

2,1
6,4
3,1
3,6
3,1
2,2
3,5
3,6
4
3,7
4,2
1,9
10
3,5
3,2
1,3
4,3
4,5
5,1
4,5
2,2
1,9
5,4
1,7
3,4
3,3
2,8
2,7
2,5
2,7
8,7
1,7
4,9
2,2
1,7
2,9
4,6
3,9
7,3
2,5
5
3,5
4
4,1
1,7
2,2
7,7
4,8
2,7
6,8

4
6,1
3,3
6
3,2
6,4
5,1
2,2
6,1
4
4,7
6,3
4,7
3,3
2,6
3,1
3,4
2,3
2,9
7,5
4,8
4,6
3,4
2,9
4,7
2
6,9
6,1
8,4
2,2
6,5
4,9
5,3
2
3,6
3,3
2,4
8,1
2,1
3,9
2,4
10,1
3,2
4,2
4,6
4,8
2,7
5,3
2,9
2,2

3,7
4,1
5,1
6,5
4,2
5,8
3,6
2,6
5,6
2
3,1
5,3
3,7
2,7
4,4
5,3
7,4
4,1
7,4
3,8
2,2
4,8
3
3,3
3,6
5,5
3,4
3,6
4,1
2,7
6,3
5
8,2
2,1
2,7
4,1
8,6
5,1
5,2
1,7
3,3
3,5
10
2,1
5,2
4,2
3,6
2,9
5,2
5,4

10
5,6
5,4
10
8,5
4,4
2,7
2,4
3,3
6,7
2,8
2,7
4,7
7,4
4,7
5,5
4,7
5,4
3,5
2,2
3,2
4,2
4,7
3,6
4
3,7
4,9
3,2
5,5
3,9
2,3
4,7
6
3,2
4,5
5,8
4,5
3,5
8,1
3,4
8,7
4,8
4,4
4
6,9
5,5
4,2
3,7
9
6,9

4,6
3,3
2,3
2,6
4,9
5,6
2
6,3
3,1
7,6
2,4
10
5,3
2,9
6,2
3
4,8
3
4,9
3,2
5,3
4,3
4,6
4,3
3,6
6,1
4,9
4,8
4,9
6,5
6,3
8,8
5
3,7
5,6
6,8
1,7
4,4
3,1
1,7
6,9
3
8,2
5,8
5,4
5,7
2,9
3,4

2,9
3,5
2,5
2,1
3,2
2,2
